# Modifying cellulose fibres with carbon dots: a promising approach for the development of antimicrobial fibres

**DOI:** 10.1098/rsos.231755

**Published:** 2024-04-17

**Authors:** Remya Radha, Zinb Makhlouf, Rasha Diab, Mohammad H. Al-Sayah

**Affiliations:** ^1^ Department of Biology, Chemistry and Environmental Sciences, American University of Sharjah, Sharjah 26666, United Arab Emirates; ^2^ Materials Science and Engineering Program, College of Arts and Sciences, American University of Sharjah, Sharjah 26666, United Arab Emirates

**Keywords:** carbon dots, cellulose, boronic acid, curcumin, antimicrobial fibres, *Staphylococcus epidermidis*

## Abstract

This study focuses on the development of antimicrobial fibres for use in medical and healthcare textile industries. Carbon dots (CDs) were designed with boronic acid groups for the attachment to cellulose fibres found in cotton textiles and to enhance their attachment to glycogens on bacterial surfaces. Boronic acid-based and curcumin-based CDs were prepared and characterized using various techniques, showing a nanoscale size and zeta potential values. The CDs inhibited the growth of both *Staphylococcus epidermidis* and *Escherichia coli* bacteria, with UV-activated CDs demonstrating improved antibacterial activity. The antimicrobial activity of the CDs was then tested, revealing strong adherence to cellulose paper fibres with no CD diffusion and potent inhibition of bacterial growth. Cytotoxicity assays on human cell lines showed no toxicity towards cells at concentrations of up to 100 µg ml^−1^ but exhibited increased toxicity at concentrations exceeding 1000 µg ml^−1^. However, CD-modified cellulose paper fibres showed no toxicity against human cell lines, highlighting the antimicrobial properties of the CD-modified cellulose fibres are safe for human use. These findings show promising potential for applications in both industrial and clinical settings.

## 1. Introduction

The recent pandemic has heightened the public awareness of healthcare safety and occupational hygiene [1,2]. Consequently, there has been increased focus on developing materials that can effectively prevent the spread of harmful microorganisms, including bacteria, fungi and viruses [3–5]. A notable example of such materials is the development of self-disinfecting fabrics and antimicrobial textiles. The textile industry has witnessed a significant increase in the demand for functional textiles driven by various sectors, including healthcare, cosmetics, hospitality, sports and the military [6].

This increase in demand has led to advancements in textile industrialization and technology, allowing various approaches to achieve antimicrobial properties. Such methods involve chemical treatments and the use of antimicrobial materials such as metal nanoparticles and metal-infused fibres [7–9].

Chemical treatments involve the application of specific antimicrobial agents on the textile surface or within its fibres [10]. Commonly used chemicals include quaternary ammonium compounds, triclosan and antimicrobial polymers. These agents disrupt the cellular structure of microorganisms and prevent their growth and reproduction. Such chemical treatments are effective and can provide long-lasting antimicrobial properties to textiles; however, there are concerns about their potential environmental and health impacts. For example, quaternary ammonium compounds leach out from fabrics because they are not chemically attached to fibres, whereas triclosan contributes to bacterial resistance and environmental contamination by dioxins [11–16].

On the other hand, metal-based materials, which have long been recognized for their antimicrobial properties, are often integrated into textiles through processes such as electrospinning or coating [17,18]. However, metals and technology are expensive and because these metals are not attached to fibres with strong chemical bonds, they are prone to leaching into the environment [19]. Therefore, there is a growing interest in incorporating textile- or cellulose-based antimicrobial materials that are more eco-friendly and sustainable with minimal side effects on human health [7,13,20].

Carbon dots (CDs) are a relatively new class of carbon-based materials with antimicrobial properties and low toxicity to mammalian cells [21–26]. These nanoparticles can be readily prepared from biological carbon sources with various functional groups on their surfaces using a simple process. Furthermore, their remarkable photophysical properties, as they emit at a wide range of wavelengths upon excitation with UV or visible light, have enabled their application in photodynamic therapy and visible-light photocatalysis [27–31].

The antibacterial activities of CDs have been evaluated in several *in vitro* and *in vivo* studies. For instance, non-modified CDs synthesized from citric acid and aminoguanidine were reported to selectively inhibit highly virulent strains of *Pseudomonas aeruginosa*, indicating their potential as effective antimicrobial agents [32], while curcumin-based CDs were shown to possess antiviral activity against porcine epidemic diarrhoea virus [33–36]. Therefore, CDs are a new class of antimicrobial agents with minimal toxicity, eco-friendliness and biological compatibility with mammalian cells [37,38].

Herein, we report the modification of cellulose fibres with boronic acid-functionalized CDs as a promising approach for developing antimicrobial fibres. We hypothesized that the presence of boronic acid groups on the surfaces of the CDs will provide two critical roles: (i) the reaction of boronic acid with diol moieties on the cellulose surface works as an anchor of the CDs to the fibres through the formation of boronate ester bonds [39–42] and (ii) a similar reaction with glycans/carbohydrates on the surface of bacterial cells will help in trapping the cells to the fibres and enhance their efficacy. Moreover, the bioactivity of the CDs is expected to be enhanced upon irradiation of the CDs and modified fibres with UV light. Two types of CDs were prepared from phenylboronic acid (BACDs) alone and in combination with curcumin (PBA-CCMCDs), a natural compound with known antimicrobial activity [33]. Both types of CDs exhibited bactericidal activity against Gram-negative and Gram-positive bacterial strains in solution, in the presence and absence of UV light (365 nm). Similar activity was observed for the CD-modified fibres against *Staphylococcus epidermidis* (SE) and *Escherichia coli* (EC) bacterial strains. While there have been a number of reports on the use of CDs as antimicrobial agents for textile modifications, most of these uses require pre-modification of the fibres with agents that can attach the CDs with electrostatic attractions and, hence, allow their release into media. This study reports a new approach for the attachment of the CDs to cellulose fibres without the pre-modification of the fibres; the CDs exhibit activity while remaining securely attached to the fabric without being released.

## 2. Material and methods

### 2.1. Materials/chemicals/bacterial strains

All chemicals, including curcumin (CCM), citric acid monohydrate, phenylboronic acid (PBA) and phosphate-buffered saline tablet capsules, were purchased from commercial suppliers Sigma-Aldrich (Baden-Wurttemberg, Germany). Paper discs with a diameter of 7 mm were crafted using Whatman filter papers (Grade 1) and 100% cotton (sterile gauze swabs from Cherry Medical Supply) was used as the substrate for the attachment of the CDs. Tryptic soy broth (Millipore) and Mueller–Hinton broth (Millipore) were used for the cultivation and bioassay of different bacterial strains. All the cell media were autoclaved before use. The antibacterial activities of the CDs were assessed against Gram-negative and Gram-positive bacteria, EC (MicroKwik Culture 155065A) and SE.

Cytotoxicity studies were conducted on both the normal cell line HBMEC (human brain microvascular endothelial cell line) and cancer cell line HCT 116 (human colorectal carcinoma cell line). Both cell lines were cultured in DMEM (Dulbecco’s modified Eagle medium, Sigma-Aldrich) containing 10% (v/v) FBS (fetal bovine serum, Sigma-Aldrich), 10% (v/v) penicillin–streptomycin (Sigma-Aldrich) (10 000 U penicillin and 10 mg ml^−1^ streptomycin), trypsin-EDTA solution (Sigma-Aldrich), Dulbecco’s phosphate-buffered saline (Sigma-Aldrich), DMSO, thiazolyl blue tetrazolium bromide (Sigma-Aldrich, USA) and 24- and 96-well tissue culture plates (Corning, USA).

### 2.2. Synthesis of the carbon dots

A hydrothermal carbonization reaction was used to synthesize BACDs from PBA in a single step using previously reported procedures [43]. Initially, 100 mg of PBA was dissolved in 10 ml of deionized water and the pH was adjusted to 9.0 using NaOH (0.5 M). The mixture was then degassed for 60 min under nitrogen gas and subjected to a reaction at 160°C for 8 h in a Teflon-lined hydrothermal synthesis reactor (BAOSISHAN). After cooling, the solution was centrifuged at 12 000 rpm for 30 min to remove large precipitates and then dialyzed against water for 24 h with deionized (DI) water being changed every 6 h. The CD solution was lyophilized and further characterized for use in bioassays.

PBA-CCMCDs were produced via a thermal reaction by combining CCM (0.3 g) and PBA (0.6 g), which were previously ground using a mortar and pestle. The dry mixture was heated to 180°C for 60 min in a thermal reactor. Then, 15 ml of DI water was mixed with the brown mixture and the resulting solution was vortexed and centrifuged at 12 000 rpm for 30 min to obtain a clear aqueous CD solution. The supernatant CD solution was collected and used for bioassays and characterization.

### 2.3. Attachment of the carbon dots to cellulose fibres

The primary components of various fabrics and papers are cellulose fibres. To prepare cotton fibres for treatment, they were washed with DI water and dried at 100°C for 2 h. Approximately 150 mg of dried cotton pieces were immersed in 1.5 ml of BACD solution at room temperature for 2 h. After soaking, the pieces were squeezed and dried at 100°C for 2 h.

Paper discs (diameter 6–7 mm) were prepared using Whatman filter paper (Grade 1). The discs were treated with a solution (20 µl) of the CDs at a concentration of 50 mg ml^−1^ in PBS (pH 7.4) and kept in a closed container at room temperature for 2 h. After the incubation period, the paper discs were washed with DI water (3 × 10 µl) and allowed to air dry before use for further analysis/assays.

### 2.4. Characterization of carbon dots and modified fibres

The structural and fluorescence properties of various CDs and modified papers/fibres were investigated. A UV–visible spectrophotometer (Shimadzu UV-1800) was used to measure the absorption spectra of different CDs and fluorescence emission profiles were recorded using a fluorescence spectrometer (FLSP920, Edinburgh Instruments). The zeta potentials of the CDs were analysed using an Anton Paar Litesizer 500. ATR and Fourier transform infrared (FTIR) spectrometers (PerkinElmer) were used to identify the functional groups in the CDs and modified fabrics. For the FTIR spectroscopy, the CDs and potassium bromide (KBr) were ground in a 1:100 ratio in an agate mortar, pressed into a pellet under high pressure for 2–3 min and documented for the pellet while considering the pure KBr signal as the background. High-resolution scanning electron microscopy (Ultra High-Resolution TESCAN-SEM, model Magna GMU with Oxford Azteclive EDS Analysis System with Ultimax 65 mm^2^ SSD-EDS detector) was used to confirm the morphological characteristics of the CDs. The CD sample solution was applied to the surface of a silica wafer and allowed to dry under ambient air for SEM analysis. The analysis was conducted at an accelerating voltage of 10 kV. Additionally, atomic force microscopy (AFM) was conducted in the non-contact mode using an hpAFM (NanoMagnetic Instruments). The CD samples were deposited on a silicon wafer for analysis. The AFM measurements were carried out in dynamic lift mode with a 100 nm lift-off distance and a scanning speed of 1.0 µm s^−1^.

The modified cotton and paper discs were characterized using several techniques. The surface morphology was analysed by scanning electron microscopy (SEM) using a VEGA3 model from TESCAN. The cotton samples were pre-coated with gold particles for analysis. The elemental compositions of the samples were determined by energy-dispersive X-ray spectroscopy (EDS, Oxford). Raman spectroscopy was performed using a WITec Confocal Raman Microscope-α 300, using a laser power of 65 mW, a wavelength (*λ*) of 785 nm and 10× magnification with an integration time of 10 s and accumulations of 10 s. Powder X-ray diffraction (XRD) data were collected using a Panalytical X’pert3 pro multipurpose diffractometer by Cu Kα radiation with a 2*θ* range of 5–50°.

Alizarin Red S (ARS) assay [44] was conducted to determine the loading of the CDs on paper discs. After paper discs were incubated with PBS solutions (20 µl) of the CDs (50 mg ml^−1^) overnight in wells of a 96-well plate, 5 µl of the exhausted CD solution was transferred to clean dry wells. This solution was serially diluted five times to a volume of 25 µl and each solution was mixed with the PBS solution of ARS (100 µM). A control solution was treated with the same process. The difference in fluorescence emission (*λ*_ex_ = 490 nm and *λ*_em_ = 590 nm) between exhausted solution and control was used to estimate the loaded CDs per paper disc.

### 2.5. Antibacterial assay

The antimicrobial properties of the CDs were initially tested against the bacterial strain SE. The bacterial cultures were grown in 10 ml autoclaved TSB medium tubes by inoculating a single colony from the corresponding plated culture on a TSA plate and incubated overnight at 37°C and 180 rpm. Freshly grown bacterial cultures were used as seed cultures in further experiments. The surface plating method (L-rod plating) was used to observe colony-forming units (CFUs) or cell viability in the presence of the CDs. The bacterial cultures were serially diluted and tested using different combinations of aqueous CD solutions and bacterial populations. Aliquots of 100 µl of CD bacterial suspension (in appropriate dilutions) were incubated for 120 min at room temperature to allow for better diffusion. The entire 100 µl suspension was uniformly surface-plated on MHA plates and the number of bacterial colonies formed on the plates was counted for all treated samples after overnight incubation at 37°C. The experiments were repeated using both UV-treated and untreated CDs to assess the effects of UV radiation on CD activation. To check the effect of UV radiation on the activation of the CDs, vials containing the CD–bacterial mixture were kept under UV light for 120 min for plating.

#### 2.5.1. Microtitre plate experiment with bacterial cells and the carbon dots

To assess the inhibitory concentrations of the CDs (IC_50_ and IC_90_) for bacterial cells, experiments were conducted in 96-well microtitre plates using a previously reported modified protocol [1]. For each bacterial strain, a seed culture was prepared and 1% of the freshly grown culture was inoculated into a new broth tube (MHB). The optical density (OD) of the broth was monitored at *A*_600_ until it reached 0.22 (1 × 10^8^ CFU ml^−1^) for use in the bioassay.

Quantification of the inhibitory concentrations of the CDs (IC_50_ and IC_90_) for each condition was performed using a plate assay with measurements taken in triplicate wells. To create a linear range of diluted concentrations, the CD aqueous solution (40 mg ml^−1^) was serially diluted with PBS (pH 7.4). Edge wells were left empty and filled with DI/PBS. In each well, 50 µl of bacterial cell suspension containing 10^6^ CFU ml^−1^ was added, resulting in a final bacterial population of 10^5^ CFU ml^−1^ (cell–CD suspension). Proper blanks (PBS/MHB), positive control (gentamicin) and cell culture control were included for each plate. The plate was maintained at room temperature for 120 min to allow better diffusion of the CDs into the cells. For UV treatment, the plates were exposed to UV light (5 W) for 90–120 min inside a Fluorescence Analysis cabinet (Spectroline, USA) using 365 nm longwave UV light at a distance of approximately 8 cm from the top surface of the plate. After the treatment, the plates were incubated overnight at 37°C. To determine the efficiency of the bactericidal function of the CDs, the viable cell numbers in the control and treated samples were compared by analysing the *A*_600_ values of the control and treated wells using a BioTek 800^TS^ microplate reader. The decrease in absorbance in the treated wells compared with that in the control wells was used to assess the bactericidal effect of the CDs. The inhibitory concentrations of the CDs (IC_50_ and IC_90_) were calculated using the plot of percentage of growth of cells versus the concentration of CDs for each condition and bacterial strain.

#### 2.5.2. Effect of contact time on bioactivity

To examine the impact of the interaction time between the CDs and the bacterial cultures, a time-dependent experiment was carried out with and without UV radiation. Bacterial cultures (5 × 10^5^ CFU ml^−1^) were incubated with CD solutions at three different concentrations (1, 5 and 10 mg ml^−1^) for different durations (2, 4 and 8 h). Following incubation, 10 µl of the diluted solutions was plated on MHB agar plates and incubated at 37°C overnight. The growth of bacteria on the agar plates was compared with that of the control solution without the CDs to assess the effectiveness of the CDs at different time intervals.

#### 2.5.3. Bioactivity of carbon dot-modified cellulose fibres (paper discs)

Cellulose fibres were modified with the CDs using the method outlined in §2.3. To evaluate the bioactivity of the modified paper discs, a microtitre plate assay was conducted using varying populations of microbial strains. Small filter paper discs with a diameter of 6–7 mm were treated with 20 µl of CD solution (50 mg ml^−1^) for 12 h, washed and dried. CD-modified discs were incubated for 2 h with serially diluted bacterial solutions (OD_600_ = 0.22; ~10^8^ CFU ml^−1^) and one of the plates was treated with UV light for 1.5–2 h in a 96-well plate. The discs were removed and the wells (100 µl) were incubated overnight at 37°C. Control plates were treated with unmodified (control) paper discs under the same conditions.

To confirm the effectiveness of CD-modified paper discs in preventing bacterial growth, an agar plate assay was conducted. The modified discs were incubated with a bacterial solution (20 µl; 5 × 10^5^ CFU ml^−1^) for 2 h with or without exposure to UV light (using a Spectroline Fluorescence Analysis cabinet) at a distance of 8 cm with 365 nm waves. After incubation, PBS (80 µl) was added to the discs and 10 µl of the solution was plated on MHB agar plates and incubated overnight at 37°C. Controls, in which discs without the CDs were used, were also used. The growth of bacteria on the plates was compared between the CD-modified discs and control discs to determine the effectiveness of the modified paper discs in preventing bacterial growth.

### 2.6. Cell culture and cell viability analysis

To assess the cytotoxicity of the CD solutions and CD-modified paper discs, the study employed both the normal cell line HBMEC and the cancer cell line HCT 116. MTT assay was used to measure the enzymatic activity of the cell cultures. A density of 50 000 cells per well in 500 μl of DMEM complete medium was used to seed the cells into 24-well plates, which were then incubated overnight at 37°C, 5% CO_2_ and a minimum of 95% specific humidity level. The medium was then replaced with 500 μl of fresh medium containing different CDs (BACDs and PBA-CCMCDs) at concentrations of 0.1, 1.0 and 10.0 mg ml^−1^ in triplicate. After 48 h of treatment, the MTT solution (5 mg ml^−1^ in PBS) was added to each well to maintain a final concentration of 0.5 mg ml^−1^ and incubated for 4 h. Then, 500 µl of DMSO was added to each well to dissolve the contents and the plate was maintained at room temperature for 10–15 min. The absorbance of each well was measured at 570 nm wavelength using a microplate reader (BioTek, TS 800). The experiment was conducted in triplicate using three different batches of the CDs. The culture viability was calculated by comparing the absorbance of the treated sample cells (wells treated with the CDs) with that of the control wells (cells not treated with the CDs).

To evaluate the cytotoxicity of the paper discs modified with BACDs and PBA-CCMCDs, the MTT protocol described above was used. Paper discs were coated with the CDs according to the protocol described in §2.3. To prevent contamination, the CDs were coated onto the paper discs in a laminar hood. Additionally, control discs (paper discs not modified with the CDs) were included in the study and were subjected to the same protocol as the modified discs.

### 2.7. Statistical analysis

Bioassay experiments were conducted in triplicate and repeated in at least three batches. Statistical analysis was performed using GraphPad Prism version 6.05 and two-way and one-way ANOVAs were used to compare data between different treatment conditions. A Bonferroni multiple comparison test was used to confirm the statistical significance of the data.

## 3. Results and discussion

We conducted a comprehensive structural characterization of all the synthesized CDs, encompassing assessments of their fluorescence and absorption properties, functional groups, charge and size and evaluation of their structural integrity and bioassays.

### 3.1. Characterization of the carbon dots

**Figure 1.**
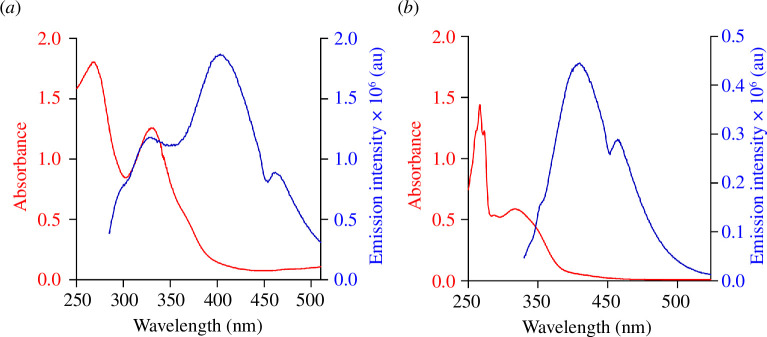
Absorption and emission properties of the CDs. Absorption and emission spectra of (*a*) BACDs and (*b*) PBA-CCMCDs. The absorption profiles of the CDs in the wavelength range of 200–500 nm are represented by the red plots. BACDs exhibited two absorption peaks at 268 nm and 329 nm (*a*, red plot). PBA-CCMCDs showed a single peak at 317 nm (*b*, red plot). The fluorescence properties of the CDs were analysed by recording their emission spectra with the aqueous solutions of the CDs in PBS excited at 270 nm (BACDs) and 315 nm (PBA-CCMCDs).

The CDs were synthesized using PBA (for BACDs) or a combination of PBA and CCM (PBA-CCMCDs) using a hydrothermal reaction [33]. The absorption profiles of the obtained CDs were determined by recording their aqueous solutions in phosphate buffer (pH 7.4), as depicted in figure 1 (red plots). BACDs exhibited absorption peaks at 268 and 329 nm (figure 1*a*, red plot), whereas PBA-CCMCDs exhibited absorption peaks at 275 and 317 nm (figure 1*b*, red plot). Meanwhile, the CDs exhibited strong fluorescence (figure 1, blue plots) in the visible region, yet the starting material, PBA derivatives, was non-fluorescent in this region [43]. BACDs exhibited the highest fluorescence emission at 403 nm when excited at 270 nm (figure 1*a*, blue plot), indicative of functional CDs, whereas PBA-CCMCDs exhibited the highest emission at 410 nm when excited at 315 nm (figure 1*b*, blue plot).

**Figure 2.**
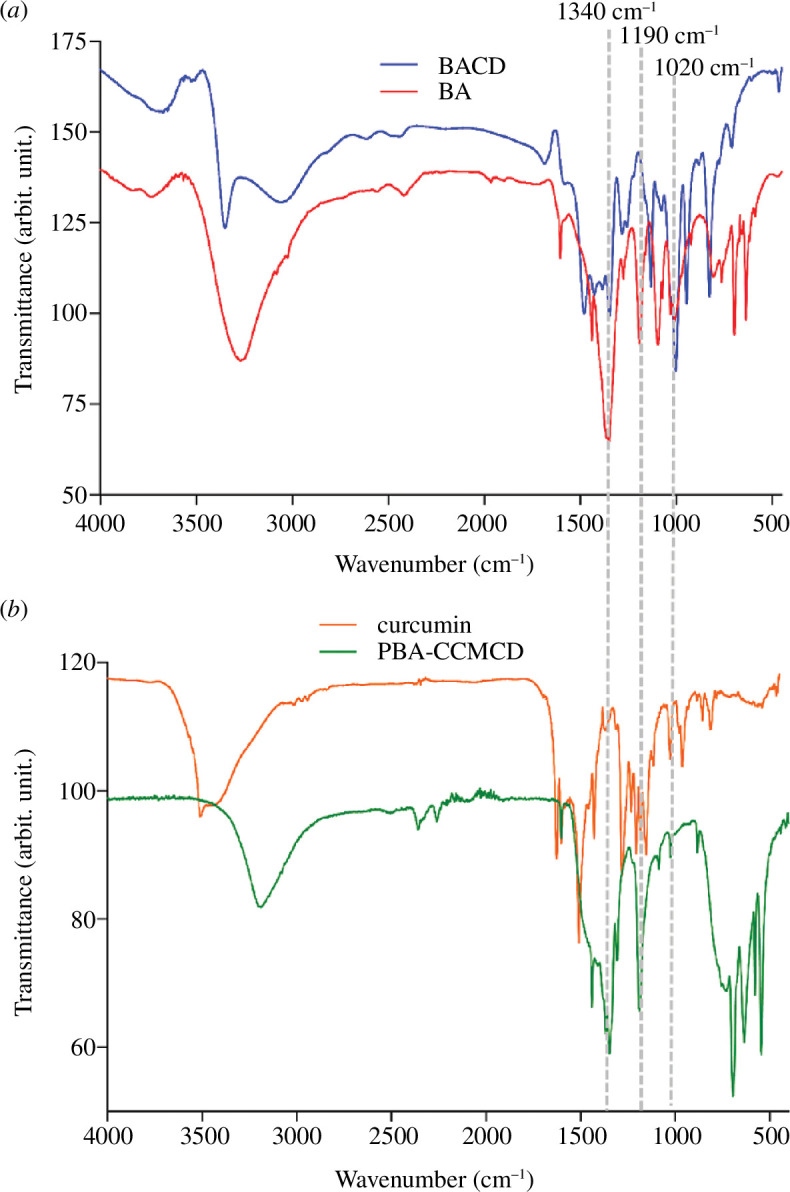
FTIR/IR spectral analysis of the CDs and raw materials. (*a*) Blue spectra correspond to BACDs and red spectra correspond to their precursor molecule PBA. (*b*) Green spectra correspond to PBA-CCMCDs and orange spectra correspond to their original molecule CCM. The signals indicated with dashed lines represent the characteristic peaks that correspond to the B–O bond.

The FTIR spectra of the BACDs exhibited characteristic peaks at approximately 3200 cm^−1^ for O—H and 1340, 1190 and 1020 cm^−1^ for B–O (figure 2*a*) [45]. The IR spectrum of CCM exhibited characteristic peaks for O–H (3500–3200 cm^−1^) and carbonyl groups (~1750 cm^−1^; figure 2*b* orange plot). The ATR spectra of PBA-CCMCDs (figure 2*b*, green plot) confirm the presence of the same characteristic peaks for O–H at ~3200 cm^−1^ and B–O at ~1340, 1190 and 1020 cm^−1^.

**Figure 3.**
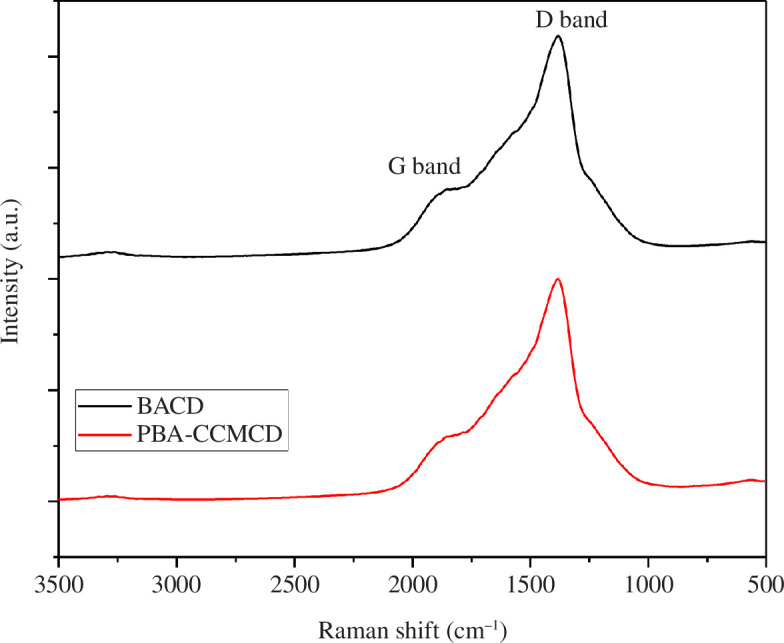
Raman spectral analysis of the CDs. The spectra show the G band and the D band of the carbon-based material.

Raman spectroscopy was carried out to investigate the inner morphology of the CDs. The Raman spectrum of the CDs (figure 3) consists of two major bands (the G and the D bands). However, the deconvolution of these spectra (electronic supplementary material, figure S1) shows three bands: D (1359 cm^−1^), G (~1480 cm^−1^) and G* (~1890 cm^−1^), which are typical for carbon polymorphs and the CDs [46]. The most intense D and G bands correspond to *sp* ^3^-hybridized amorphous carbon and the stretching mode of the *sp* ^2^ carbon network, respectively [47,48]. The G* band is attributed to the presence of boron–carbide stretching within the *sp* ^3^-hybridized amorphous carbon [49,50].

**Figure 4.**
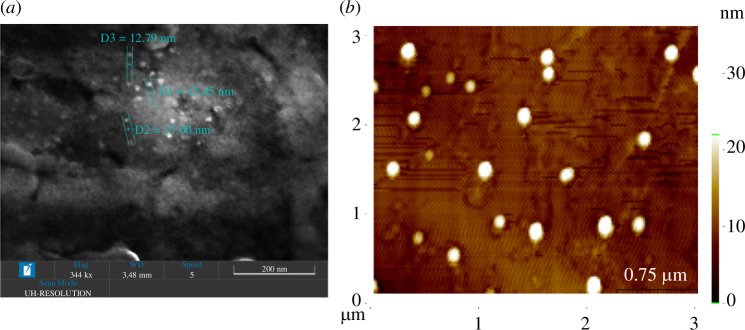
SEM and AFM images of the CDs. (*a*) Ultrahigh-resolution SEM image of BACDs and (*b*) AFM image of BACDs. For the AFM scanning, the CDs were dissolved in PBS (0.05 mg ml^−1^) and uniformly coated on silicon wafer supports.

Zeta potential is a crucial parameter for predicting the level of electrostatic repulsion between similarly charged particles in a dispersed system [51]. Notably, BACDs exhibited a zeta potential of −33.7 mV, while PBA-CCMCDs exhibited a zeta potential of 16.5 mV at a pH of 7.4. Additionally, we conducted a high-resolution SEM analysis of BACDs, revealing a size distribution ranging from 10 to 50 nm in diameter (a sample image is depicted in figure 4*a*). To further confirm the presence of nano-sized CD molecules, we used AFM for both BACDs (figure 4*b*) and PBA-CCMCDs (electronic supplementary material, figure S2).

### 3.2. Characterization of carbon dot-modified cellulose fibres

Paper- and cotton-based materials are gaining growing interest in a wide range of applications owing to their inherent advantages such as widespread availability, affordability and eco-friendliness [52]. The CDs were attached to cellulose fibres (cotton or paper) by direct incubation of the fibres with the CD solution at neutral pH, as detailed in §2.3. The reaction of PBA groups with diols on the cellulose surface to form boronate esters leads to the attachment of the CDs to the surface [53]. The fibres were then washed several times to remove the unreacted CDs.

The attachment of the CDs to the fibres can be visibly observed by comparing the fluorescence colours of the modified and unmodified fibres under UV light (electronic supplementary material, figure S3). Additionally, ARS assay [44] was used to estimate the loading of BACDs on paper discs. The binding of PBA groups to ARS enhances the fluorescence of the dye. Thus, after the paper discs were soaked in BACD solution (50 mg ml^−1^), the concentration of the remaining CDs solution was measured by the emission difference of CD–ARS mixture before and after soaking. The results showed that ~30% of the CDs are loaded on the discs which accounts for ~0.3 mg of CDs loaded per paper disc. The loading was also visually noticeable by the change in the flourescence of CD-modified discs with ARS versus non-modified discs (electronic supplementary material, figure S5).

**Figure 5.**
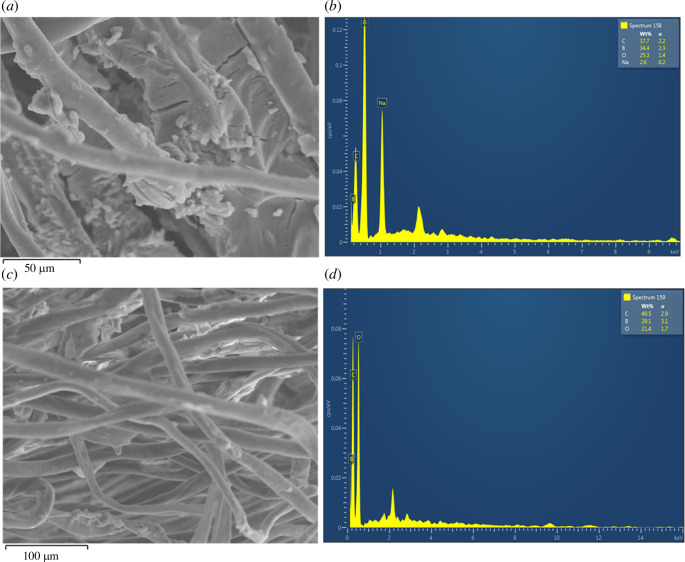
SEM/EDX images of BACD- and PBA-CCMCD-attached fibres. (*a*) SEM image and (*b*) EDX spectrum of BACD-coated cotton. (*c*) SEM and (*d*) EDX images for PBA-CCMCD-modified cotton.

Furthermore, SEM/EDX analyses were conducted to confirm the attachment of the CDs to the fibres. The SEM images of the BACD- and PBA-CCMCD-modified cotton fibres (figure 5) and the EDX signals of the elemental distribution of the fibre surfaces indicate the presence of B atoms corresponding to the presence of the CDs on the surface. As shown in the electronic supplementary material, figure S4, XRD analysis reveals the crystalline nature of both synthesized CDs, with sharp diffraction peaks observed at various Bragg angles. However, the XRD data demonstrate that the inclusion of the CDs did not result in significant modifications to the diffraction pattern of fibres, implying that the fibre framework experienced minimal to no changes.

### 3.3. Antibacterial activities of the carbon dots

The bactericidal functions of BACDs against different microbial strains were initially screened under varying concentrations of BACDs and other physical conditions, as described in §2.5, using a surface plating method. Significant bactericidal effects were observed in CD-treated plates, especially in UV-treated plates (electronic supplementary material, table S1). Electronic supplementary material, figure S6, shows the reduction of SE cells upon treatment with BACDs for 1.5 h at room temperature along with the UV-treated samples. The relative CFU reduction (the number of viable cells) under each condition is presented in electronic supplementary material, table S1. Incubation with BACDs substantially inhibited SE growth. Furthermore, when BACDs were irradiated with low-intensity UV light (365 nm), SE growth was significantly reduced, providing additional evidence of the pronounced bactericidal effect, particularly when BACDs were combined with UV light.

**Figure 6.**
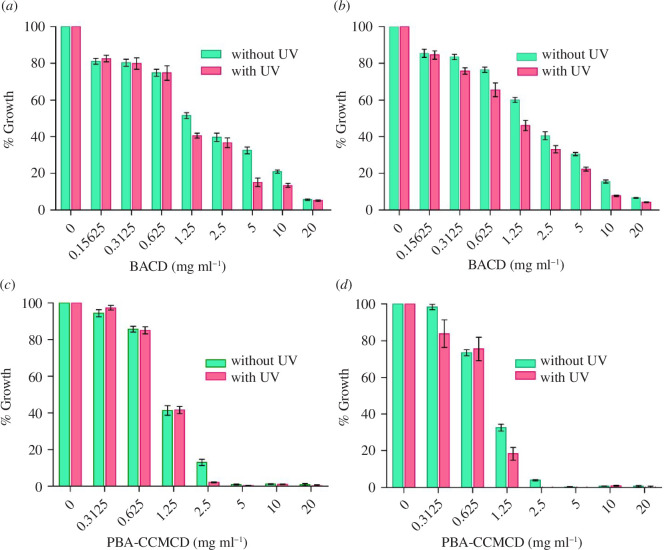
Antibacterial assays with original solutions of the CDs. (*a*) Against SE with BACDs and (*b*) against EC with BACDs. Antibacterial assays with PBA-CCMCDs against (*c*) SE and (*d*) EC.

However, to calculate the minimum inhibitory concentration (MIC) of the CDs, which is the lowest concentration required to inhibit bacterial growth by 90%, microdilution assays were conducted in 96-well plates. Both types of CDs were tested against the bacterial strains in the presence and absence of UV light. Figure 6 shows the relative growth of two bacterial strains SE and EC in the presence of various concentrations of the CDs as compared to the control (no CDs).

The results showed that the efficiency of bacterial growth inhibition for both Gram-negative and Gram-positive bacteria was slightly higher under UV irradiation (figure 6). BACDs showed a gradual increase in bacterial growth inhibition as the concentration of BACDs increased from 0 to 20 mg ml^−1^ in both SE (figure 6*a*) and EC (figure 6*b*). However, for PBA-CCMCDs (figure 6*c,d*), very high bacterial inhibition was observed for concentrations larger than 2.5 mg ml^−1^.

The MIC data obtained (table 1) showed that PBA-CCMCDs were more effective at inhibiting growth at lower concentrations than BACDs. These results suggest that the combination of CCM and PBA leads to a more effective CD, especially since the CDs made from CCM alone (CCMCDs) were less effective than PBA-CCMCDs (electronic supplementary material, figure S7). Accordingly, it can be argued that the presence of PBA enhanced the binding of PBA-CCMCDs to surface bacteria, which made them more effective. In addition, zeta potential measurements indicate that PBA-CCMCDs are positively charged, which makes their electrostatic binding to the negatively charged bacterial surfaces more favoured than that of BACDs (−33 mV) [54,55].

**Table 1.** Bactericidal effect of different CDs.

type of CDs		without UV		with UV	
		**EC**	SE	**EC**	SE
BACDs	IC_50_ (mg ml^−1^)	1.8	1.2	0.9	0.91
	IC_90_ (mg ml^−1^)	16.3	16.7	9.7	14.9
PBA-CCMCDs	IC_50_ (mg ml^−1^)	1	1	0.7	1
	IC_90_ (mg ml^−1^)	2.2	3.3	1.8	2.2

Furthermore, to investigate the effect of the incubation time of bacteria with the CDs on their antibacterial activity, bacterial cultures were incubated with CD solutions (BACDs and PBA-CCMCDs) at different concentrations (1, 5 and 10 mg ml^−1^) with and without UV irradiation. For the UV-treated samples, the CD–bacterial solutions were initially treated with UV for 2 h (for all samples). Aliquots were taken at 2, 4 and 8 h intervals, diluted 10 times and plated on MHB agar for overnight growth at 37°C. The growth of the bacteria was compared to that of the control solution without the CDs (electronic supplementary material, figure S8).

The results (table 2) show that the presence of UV light enhances the bioactivity of the CDs; however, this is more crucial for BACDs. Even after 8 h of incubation at 10 mg ml^−1^, the BACDs showed similar growth to the control in the absence of UV light; however, under the same conditions, PBA-CCMCDs were more effective against both bacteria. Accordingly, BACDs were more effective under UV light at lower concentrations than PBA-CCMCDs for both bacteria after short incubation times. Therefore, it can be inferred that a longer incubation time is more crucial for the activity of PBA-CCMCDs than that of BACDs.

**Table 2.** Time-based antibacterial assay: a comparison between the control and the samples.

	BACDs						PBA-CCMCDs					
	1 **mg ml^−1^**		5 **mg ml^−1^**		10 **mg ml^−1^**		1 **mg ml^−1^**		5 **mg ml^−1^**		10 **mg ml^−1^**	
time (h)	+UV	−UV	+UV	−UV	+UV	−UV	+UV	−UV	+UV	−UV	+UV	−UV
	**EC**											
2	+++	+++	+	+++	‒	+++	+++	+++	+	+++	‒	+++
4	+++	+++	‒	+++	‒	+++	+++	+++	+	+++	‒	++
8	+++	+++	‒	+++	‒	+++	++	+++	+	++	‒	‒
	**SE**											
2	+++	+++	‒	+++	‒	+++	+++	+++	++	+++	‒	+++
4	+++	+++	‒	+++	‒	+++	+++	+++	++	+++	‒	++
8	++	+++	‒	+++	‒	+++	++	+++	+	+++	‒	+

(+++) = Similar growth to the control; (−) = no growth.

### 3.4. Antibacterial properties of the modified cellulose paper fibres

**Figure 7.**
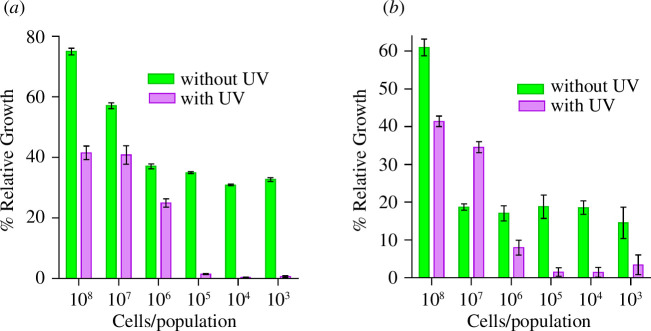
Paper disc bacterial assay: BACD. (*a*) SE and (*b*) EC. The UV treatment was found to be extremely significant with a *p*-value of ˂0.0001 for SE and EC.

Once the CDs were shown to be active in solution, the CD-modified paper fibres were tested against the same bacterial strains. ATTCC 147 test [56] of BACD- and PBA-CCMCD-modified paper fibres against bacterial strains (SE and EC) (electronic supplementary material, figure S9) showed that the CDs do not diffuse from the paper discs, most likely due to the formation of the borate ester bond. Hence, to investigate the efficacy of the modified paper fibres, paper discs (6–7 mm in diameter) were incubated with the CD solutions (20 µl, 50 mg ml^−1^) for 2 h. After washing and drying, each dry disc was placed in a well of a 96-well microtitre plate and incubated with bacterial solutions (50 µl) at different CFU counts (as outlined in §2.5). After 2 h of incubation, the discs were removed and the plates were incubated overnight. Figure 7 depicts the growth of bacteria SE and EC when incubated with BACD-modified paper fibres relative to the controls at each bacterial dilution. The results showed that when combined with UV light, BACD-modified paper fibres reduced SE growth by more than 98% with 10^5^ CFU ml^−1^ (figure 7*a*) and more than 90% bacteria with 10^6^ CFU ml^−1^ of EC bacterial strain (figure 7*b*). These results indicate that the CDs retained their antibacterial activity when attached to the surface and this activity was enhanced upon illumination with UV light. The large difference in the enhancement by the presence of UV for the CDs on paper fibres versus solution can be attributed to the diffraction of UV by the fibres, which leads to higher absorption/exposure by the CDs; in solution, a significant portion of UV light passes through without diffraction.

**Figure 8.**
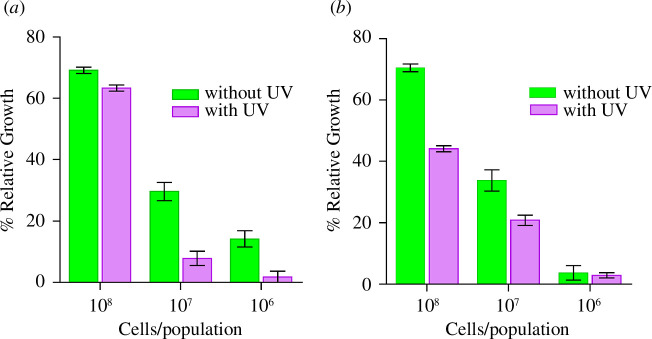
Paper disc bacterial assay: PBA-CCMCDs. (*a*) SE and (*b*) EC. The UV treatment was found to be significantly effective with a *p*-value of 0.0010 for SE and 0.0002 for EC.

Cellulose fibres in paper discs modified with PBA-CCMCDs exhibited higher efficacy than paper fibres modified with BACDs against SE and EC bacterial strains. A relative growth plot for the bacterial strains SE and EC on PBA-CCMCD paper discs is presented in figure 8. The results indicated that the PBA-CCMCD-modified paper discs led to a reduction in growth by more than 98% for SE and 90% for EC at 10^6^ CFU ml^−1^ in the presence of UV light (figure 8). To test the effect of PBA on the efficacy of the CDs and modified paper fibres, we also prepared the CCMCDs without PBA and incubated paper discs with these CDs. After washing, the antibacterial activity of the CCMCD-treated discs was tested under conditions similar to those of PBA-CCMCDs. CCMCDs exhibited high potency against both bacterial strains (electronic supplementary material, figure S7) in solution but the CCMCD-treated paper discs showed no activity against the bacterial strains. These results highlight the importance of PBA as an anchoring group of the CDs to the surface of the fibres and, hence, the induced bioactivity of cellulose.

**Figure 9.**
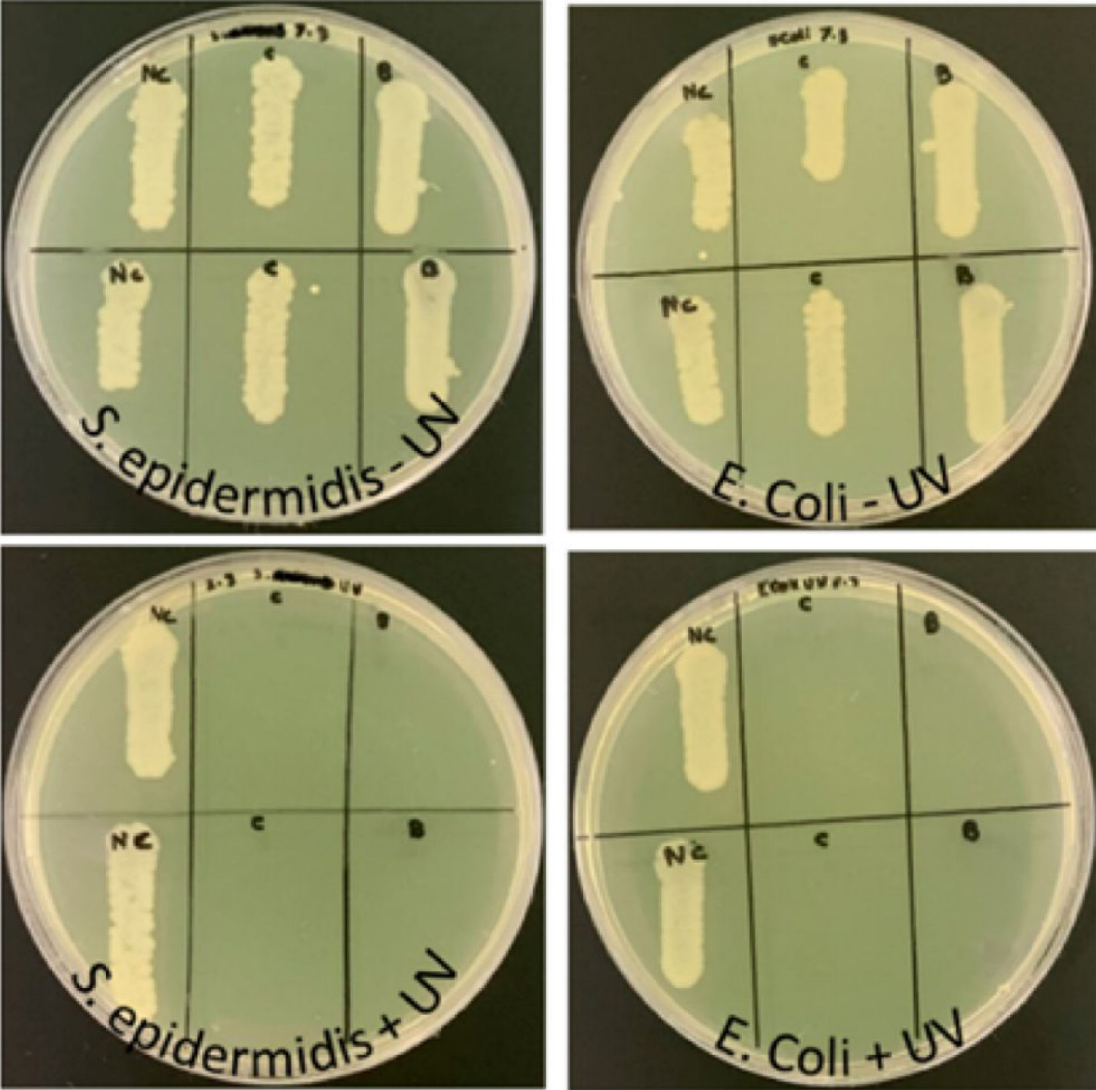
Paper disc assay on agar plates. EC or SE was incubated without UV for 2 h with NC: control disc, B: BACD disc and C: PBA-CCMCD disc.

Additionally, we conducted tests on MHB agar plates to evaluate the bactericidal properties of the modified paper discs. The modified paper discs, along with the control paper discs, were incubated for 2 h with EC or SE bacteria (20 µl; 5 × 10^5^ CFU ml^−1^) with or without UV exposure. Subsequently, the discs were washed with PBS and 10 µl of the washed solution was plated on MHB agar plates and incubated overnight at 37°C. Washed discs were plated on MHB agar plates under similar conditions. Figure 9 shows the growth of bacteria in the wash solutions of the paper discs. This figure shows that the washed solutions of the fibres incubated in the absence of UV light had bacterial growth similar to that of the control (unmodified paper discs). Meanwhile, the washed solutions of the modified paper discs incubated with the bacteria in the presence of UV light completely inhibited the growth of both bacterial strains. These results suggest that the modified fibres possessed significant antibacterial properties in the presence of UV light and/or a significant ability to bind the bacteria tightly to the fibre, such that none was observed in the washed solution. However, upon comparing the bacterial growth on the washed discs (electronic supplementary material, figure S10), it was clear that the absence of bacteria from the washed solution of the UV-activated disc was mainly due to the bactericidal activity of the discs, as minimal bacterial growth was observed around the paper discs. These results indicate that the presence of UV light is crucial for the activity of the CDs on the cellulose surface. These bioactivity results are consistent with previous studies on CD-modified textiles [57] and the CDs immobilized on synthetic polymer surfaces, suggesting the formation of reactive oxygen species as the mechanism for the bactericidal activity of CDs [58].

#### 3.4.1. Cytotoxicity assays

To assess the safety of the fibres, the cytotoxicity of the CDs and modified fibres was evaluated against HBMEC and HCT 116.

**Figure 10.**
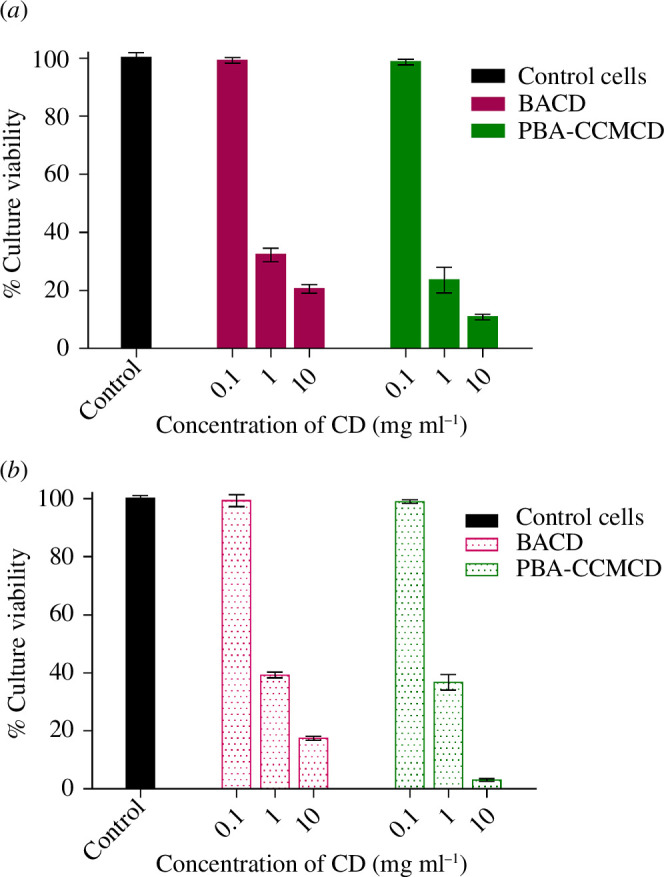
Cell culture viability with the CDs: (*a*) HBMEC and (*b*) HCT 116. The cells were grown in 24-well plates (5 × 10^4^ cells per well) in a complete DMEM. After overnight incubation, the cells were treated with various levels of the CDs (0.1, 1 and 10 mg ml^−1^), incubated for 72 h and cell viability was evaluated by using MTT. The results, expressed as a percentage of viability, are the means of three independent experiments with different batches of the CDs in triplicate. The wells without being treated with the CDs were considered control cells.

**Figure 11.**
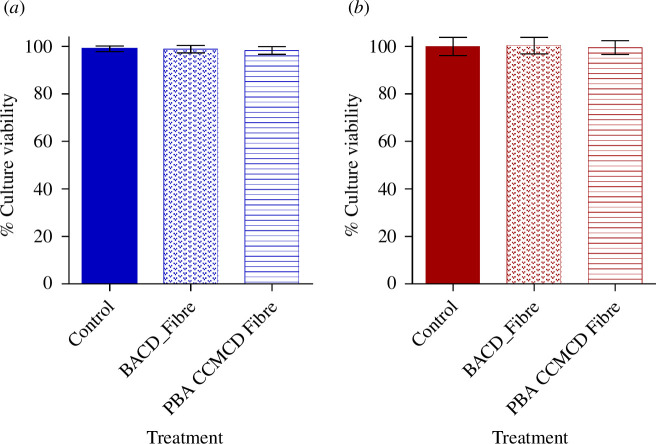
Cytotoxicity studies with modified paper discs with cell lines (*a*) HBMEC and (*b*) HCT 116. The data represent the percentage of cell viability following treatment with CD-modified fibres, gathered from three distinct experiments.

As shown in figure 10, the viability of both cell lines decreased in a dose-dependent manner with increasing CD concentration. However, the incubation of HBMEC normal cells (figure 10*a*) and cancer cell line HCT-116 (figure 10*b*) with BACDs and PBA-CCMCDs at a concentration of 0.1 mg ml^−1^ (100 μg ml^−1^) did not lead to a decrease in cell viability. Higher concentrations (>1 mg ml^−1^) significantly reduced the number of viable cells. These results suggest that the functional CDs can be safely used in human applications within a certain concentration range. However, cytotoxicity assays conducted on the modified paper discs (BACDs and PBA-CCMCDs) against HBMEC (figure 11*a*) and HCT 116 (figure 11*b*) showed no evidence of toxicity in these cell lines. This indicated that anchoring the CDs to the fibre surface decreased their cytotoxicity while maintaining their antibacterial activity. Similar results with low toxicity of the CDs against mammalian cells and high toxicity to bacteria were reported before for CDs immobilized on polymer surfaces [58]; this can be attributed to the different mechanisms of toxicity against the two cell types.

## 4. Conclusions

This study describes the bactericidal properties of the CDs synthesized from PBA and CCM. It was found that the concentration of the CDs and UV treatment were important experimental factors that affected the effectiveness of the antimicrobial properties of the CDs. Interestingly, exposure to UV light enhanced the antimicrobial effectiveness of these CDs, supporting the photodynamic mechanism of the bioactivity of the CDs. The study also successfully modified cellulose fibres in paper discs using these CDs and the modified paper fibres were tested for antimicrobial activity against SE and EC. The modified paper fibres were found to be effective against these bacteria and non-toxic to human cell lines. The successful conjugation of the CDs with cellulose in paper discs, along with their antimicrobial properties and biocompatibility, holds great promise for the ability to modify cellulose fibres in textiles. These findings could pave the way for the development of innovative materials with enhanced functionality, leading to improved antimicrobial textile products.

## Data Availability

Data are available from the Dryad Digital Repository [59]. Electronic supplementary material is available online [60].
